# 7-Methyl-5,6,7,8-tetra­hydro-1-benzo­thieno[2,3-*d*]pyrimidin-4-amine

**DOI:** 10.1107/S1600536811021155

**Published:** 2011-06-11

**Authors:** Mohamed Ziaulla, Afshan Banu, Noor Shahina Begum, Shridhar I. Panchamukhi, I. M. Khazi

**Affiliations:** a Department of Studies in Chemistry, Bangalore University, Bangalore 560 001, Karnataka, India; b Department of Studies in Chemistry, Bangalore University, Bangalore 560 001, India; c Department of Chemistry, Karnatak University, Dharwad 580 003, India

## Abstract

In the title compound, C_11_H_13_N_3_S, two of the C atoms of the cyclo­hexene ring and the methyl group attached to it are disordered over two sets of sites in a 0.544 (2):0.456 (2) ratio. The benzothiene and pyrimidine rings are almost coplanar with an angular tilt of 2.371 (9)° between them. The thio­phene ring is essentially planar (r.m.s. deviation 0.05 Å), while the cyclo­hexene ring in both the major- and minor-occupancy conformers adopts a half-chair conformation. In the crystal structure, pairs of intermolecular N—H⋯N hydrogen bonds involving the amino groups result in centrosymmetric head-to-head dimers about inversion centres, corresponding to an *R*_2_^2^(8) graph-set motif. Further, N—H⋯N hydrogen bonding generates a two-dimensional hydrogen-bonded network perpendicular to the *ac* plane and running along the diagonal of the *ac* plane.

## Related literature

For the preparation of the title compound, see: Shetty *et al.* (2009 ▶). For medicinal background, see: Brown (1983 ▶); Heildelberg & Arafield (1963 ▶); De Clercq (1986*a* ▶,*b* ▶); Sishoo *et al.* (1983 ▶). For related structures, see: Akkurt *et al.* (2008 ▶); Harrison *et al.* (2006 ▶). For graph-set notation, see: Bernstein *et al.* (1995 ▶).
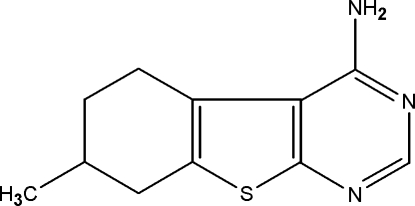


## Experimental

### 

#### Crystal data

C_11_H_13_N_3_S
*M* *_r_* = 219.30
Monoclinic, 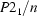

*a* = 10.395 (4) Å
*b* = 8.422 (3) Å
*c* = 13.155 (5) Å
β = 110.015 (6)°
*V* = 1082.0 (7) Å^3^
*Z* = 4
Mo *K*α radiation
μ = 0.27 mm^−1^
*T* = 123 K
0.18 × 0.16 × 0.16 mm

#### Data collection

Bruker SMART APEX CCD detector diffractometer
Absorption correction: multi-scan (*SADABS*; Bruker, 1998 ▶) *T*_min_ = 0.953, *T*_max_ = 0.958
6268 measured reflections
2347 independent reflections
1917 reflections with *I* > 2σ(*I*)
*R*_int_ = 0.044

#### Refinement

*R*[*F* ^2^ > 2σ(*F* ^2^)] = 0.053
*wR*(*F* ^2^) = 0.169
*S* = 0.84
2347 reflections
168 parameters
H-atom parameters constrained
Δρ_max_ = 0.71 e Å^−3^
Δρ_min_ = −0.41 e Å^−3^

### 

Data collection: *SMART* (Bruker, 1998 ▶); cell refinement: *SMART*; data reduction: *SAINT* (Bruker, 1998 ▶); program(s) used to solve structure: *SHELXS97* (Sheldrick, 2008 ▶); program(s) used to refine structure: *SHELXL97* (Sheldrick, 2008 ▶); molecular graphics: *ORTEP-3* (Farrugia, 1999 ▶) and *CAMERON* (Watkin *et al.*, 1996 ▶); software used to prepare material for publication: *WinGX* (Farrugia, 1999 ▶).

## Supplementary Material

Crystal structure: contains datablock(s) global, I. DOI: 10.1107/S1600536811021155/ds2116sup1.cif

Structure factors: contains datablock(s) I. DOI: 10.1107/S1600536811021155/ds2116Isup2.hkl

Supplementary material file. DOI: 10.1107/S1600536811021155/ds2116Isup3.cml

Additional supplementary materials:  crystallographic information; 3D view; checkCIF report

## Figures and Tables

**Table 1:** Hydrogen-bond geometry (Å, °)

*D*—H⋯*A*	*D*—H	H⋯*A*	*D*⋯*A*	*D*—H⋯*A*
N1—H1*A*⋯N2^i^	0.86	2.13	2.992 (3)	175
C7*A*—H7*A*⋯N2^ii^	0.98 (1)	2.47 (1)	3.400 (7)	158
C7*B*—H7*B*⋯S1^iii^	0.98 (1)	2.69 (1)	3.649 (1)	165

Symmetry codes: (i) 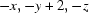; (ii) 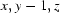; (iii) 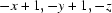.

**Fig. 1.**
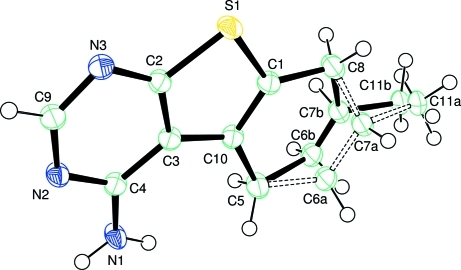
ORTEP-3 (Farrugia, 1999) view of the title compound, showing 50% probability ellipsoids and the atom numbering scheme.

**Fig. 2.**
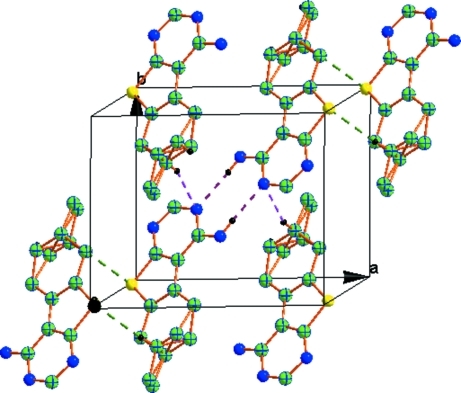
A unit cell packing of the title compound showing intermolecular interactions with dotted lines. H atoms not involved in hydrogen bonding have been excluded.

## supplementary crystallographic information

### Comment

The pyrimidine ring is a frequent partner in polycyclic heterocyclic systems of biological significance (Brown, 1983). Many potential drugs have been modelled on these compounds, particularly in cancer and virus research (Heildelberg & Arafield, 1963; De Clercq, 1986*a*,*b*). These derivatives have been reported to possess analgesic, antipyretic, antianaphilactic and antiinflammatory activities. Also, some are clinically effective antiallergic, potentially antineoplastic agents, or have significant hypocholesterolemic activity (Sishoo *et al.*, 1983). In the title compound, the fused Benzothieno and pyrimidine rings are substituted with amino and methyl groups. The C atoms C6, C7 and C11 are disordered over two sites (C6A/C6B, C7A/C7B and C11A/C11B) with site occupancy factors 0.544 (2) and 0.456 (2) resulting in minor and major conformers. The thiophene ring is essentially planar. The cyclohexene rings in both conformers is in a half-chair conformation with C7A and C7B 0.549 (4) and 0.506 (6) Å, respectively, displaced on the opposite sides from the plane formed by the rest of the ring C-atoms. In several benzothiophene derivatives the cyclohexyl ring adopts half-chair conformation (Akkurt *et al.*, 2008; Harrison *et al.*, 2006). The crystal structure is stabilized by two types of N—H···N intermolecular interactions (Table 1); N1—H1A···N2 hydrogen bonds forms centrosymmetric head-to-head dimers about inversion centres, corresponding to an *R*^2^_2_(8) graph-set motif (Bernstein *et al.*, 1995) while C7A—H7A···N2 hydrogen bonds generates two-dimensional hydrogen bonded network perpendicular to ac plane and running along the diagonal of ac plane (Fig. 2).

### Experimental

The title compound was synthesized by following the procedure reported earlier (Shetty *et al.*, 2009).

### Refinement

The bond distances of minor component of the disordered cyclohexene ring and the methyl group was restrained to C5—C6A = 1.489 (8); C6A—C7A = 1.424 (9); C7A—C8 = 1.503 (6); C7A—C11A = 1.556 (2) Å. The occupancies were refined individually for the C atoms C6, C7 and C11, the disordered atoms were grouped in Part 1 and Part 2 as Part 1: C6A, C7A and C11A with partial occupancy of 0.544 and part 2: C6B C7B and C11B with partial occupancy 0.456. In this way the occupancy disordered was modeled using the part command in *SHELXL97*. The H atoms were placed at calculated positions in the riding model approximation with N—H = 0.86 and C—H = 0.98 Å, and *U*_iso_(H) = 1.2*U*_eq_(N/C).

### Crystal data

**Table:**

C_11_H_13_N_3_S	*F*(000) = 464
*M**_r_* = 219.30	*D*_x_ = 1.346 Mg m^−^^3^
Monoclinic, *P*2_1_/*n*	Mo *K*α radiation, λ = 0.71073 Å
Hall symbol: -P 2yn	Cell parameters from 2347 reflections
*a* = 10.395 (4) Å	θ = 2.2–27.0°
*b* = 8.422 (3) Å	µ = 0.27 mm^−^^1^
*c* = 13.155 (5) Å	*T* = 123 K
β = 110.015 (6)°	Block, yellow
*V* = 1082.0 (7) Å^3^	0.18 × 0.16 × 0.16 mm
*Z* = 4	

### Data collection

**Table:**

Bruker SMART APEX CCD detector diffractometer	2347 independent reflections
Radiation source: Enhance (Mo) X-ray Source	1917 reflections with *I* > 2σ(*I*)
graphite	*R*_int_ = 0.044
ω scans	θ_max_ = 27.0°, θ_min_ = 2.2°
Absorption correction: multi-scan (*SMART*; Bruker, 1998) [is this correct?]	*h* = −11→13
*T*_min_ = 0.953, *T*_max_ = 0.958	*k* = −10→10
6268 measured reflections	*l* = −16→6

### Refinement

**Table:**

Refinement on *F*^2^	Primary atom site location: structure-invariant direct methods
Least-squares matrix: full	Secondary atom site location: difference Fourier map
*R*[*F*^2^ > 2σ(*F*^2^)] = 0.053	Hydrogen site location: inferred from neighbouring sites
*wR*(*F*^2^) = 0.169	H-atom parameters constrained
*S* = 0.84	*w* = 1/[σ^2^(*F*_o_^2^) + (0.0936*P*)^2^ + 3.4562*P*] where *P* = (*F*_o_^2^ + 2*F*_c_^2^)/3
2347 reflections	(Δ/σ)_max_ < 0.001
168 parameters	Δρ_max_ = 0.71 e Å^−^^3^
0 restraints	Δρ_min_ = −0.41 e Å^−^^3^

### Special details

**Table:**

Geometry. All s.u.'s (except the s.u. in the dihedral angle between two l.s. planes) are estimated using the full covariance matrix. The cell s.u.'s are taken into account individually in the estimation of s.u.'s in distances, angles and torsion angles; correlations between s.u.'s in cell parameters are only used when they are defined by crystal symmetry. An approximate (isotropic) treatment of cell s.u.'s is used for estimating s.u.'s involving l.s. planes.
Refinement. Refinement of *F*^2^ against ALL reflections. The weighted *R*-factor *wR* and goodness of fit *S* are based on *F*^2^, conventional *R*-factors *R* are based on *F*, with *F* set to zero for negative *F*^2^. The threshold expression of *F*^2^ > 2σ(*F*^2^) is used only for calculating *R*-factors(gt) *etc*. and is not relevant to the choice of reflections for refinement. *R*-factors based on *F*^2^ are statistically about twice as large as those based on *F*, and *R*-factors based on ALL data will be even larger.

### Fractional atomic coordinates and isotropic or equivalent isotropic displacement parameters (Å^2^)

**Table:**

	*x*	*y*	*z*	*U*_iso_*/*U*_eq_	Occ. (<1)
C1	0.3496 (3)	0.4303 (3)	0.0551 (2)	0.0240 (6)	
C2	0.3466 (3)	0.6871 (3)	0.1469 (2)	0.0227 (6)	
C3	0.2341 (3)	0.6672 (3)	0.0522 (2)	0.0221 (5)	
C4	0.1409 (3)	0.7972 (3)	0.0259 (2)	0.0225 (5)	
C5	0.1329 (3)	0.4538 (3)	−0.1015 (2)	0.0307 (7)	
H5A	0.0422	0.4744	−0.0991	0.037*	
H5B	0.1411	0.5100	−0.1633	0.037*	
C6A	0.1477 (10)	0.2803 (11)	−0.1164 (9)	0.036 (3)	0.465 (19)
H6A1	0.0699	0.2298	−0.1050	0.043*	0.465 (19)
H6A2	0.1358	0.2654	−0.1922	0.043*	0.465 (19)
C7A	0.2648 (6)	0.1894 (7)	−0.0569 (5)	0.0224 (17)	0.544 (16)
H7A	0.2274	0.1383	−0.0061	0.027*	0.544 (16)
C11A	0.3087 (17)	0.037 (2)	−0.1037 (13)	0.029 (3)	0.50 (5)
H11A	0.2289	−0.0152	−0.1514	0.044*	0.50 (5)
H11B	0.3565	−0.0329	−0.0455	0.044*	0.50 (5)
H11C	0.3679	0.0661	−0.1429	0.044*	0.50 (5)
C6B	0.1995 (8)	0.3109 (6)	−0.1466 (5)	0.021 (2)	0.535 (19)
H6B1	0.1271	0.2331	−0.1743	0.025*	0.535 (19)
H6B2	0.2169	0.3534	−0.2092	0.025*	0.535 (19)
C7B	0.2932 (8)	0.2411 (11)	−0.1011 (8)	0.035 (3)	0.456 (16)
H7B	0.3552	0.2922	−0.1329	0.042*	0.456 (16)
C11B	0.3192 (18)	0.072 (3)	−0.127 (2)	0.042 (4)	0.50 (5)
H11D	0.2934	0.0007	−0.0809	0.063*	0.50 (5)
H11E	0.4147	0.0589	−0.1166	0.063*	0.50 (5)
H11F	0.2661	0.0496	−0.2015	0.063*	0.50 (5)
C8	0.3857 (3)	0.2702 (3)	0.0241 (2)	0.0298 (6)	
H8A	0.4201	0.2045	0.0883	0.036*	
H8B	0.4580	0.2812	−0.0063	0.036*	
C9	0.2746 (3)	0.9236 (3)	0.1821 (2)	0.0258 (6)	
H9	0.2863	1.0124	0.2263	0.031*	
C10	0.2379 (3)	0.5173 (3)	0.0000 (2)	0.0231 (6)	
N1	0.0301 (2)	0.8031 (3)	−0.06281 (18)	0.0273 (5)	
H1A	−0.0235	0.8840	−0.0745	0.033*	
H1B	0.0121	0.7260	−0.1084	0.033*	
N2	0.1624 (2)	0.9225 (3)	0.09383 (18)	0.0244 (5)	
N3	0.3714 (2)	0.8140 (3)	0.21457 (18)	0.0250 (5)	
S1	0.45432 (7)	0.52481 (9)	0.17184 (5)	0.0273 (2)	

### Atomic displacement parameters (Å^2^)

**Table:**

	*U*^11^	*U*^22^	*U*^33^	*U*^12^	*U*^13^	*U*^23^
C1	0.0243 (13)	0.0236 (13)	0.0217 (13)	0.0006 (10)	0.0046 (10)	0.0007 (10)
C2	0.0237 (12)	0.0205 (13)	0.0204 (12)	0.0013 (10)	0.0028 (10)	0.0030 (10)
C3	0.0232 (13)	0.0226 (13)	0.0174 (12)	−0.0003 (10)	0.0032 (10)	0.0014 (10)
C4	0.0237 (12)	0.0210 (13)	0.0189 (12)	0.0002 (10)	0.0022 (10)	0.0024 (10)
C5	0.0312 (15)	0.0245 (14)	0.0254 (14)	0.0031 (11)	−0.0043 (12)	−0.0028 (11)
C6A	0.030 (4)	0.036 (4)	0.039 (5)	−0.004 (3)	0.008 (4)	−0.017 (3)
C7A	0.023 (3)	0.019 (3)	0.024 (3)	0.003 (2)	0.006 (2)	0.005 (2)
C11A	0.022 (4)	0.017 (5)	0.046 (6)	0.000 (3)	0.009 (3)	0.001 (4)
C6B	0.021 (4)	0.016 (2)	0.020 (3)	0.001 (2)	0.000 (2)	−0.0016 (19)
C7B	0.035 (4)	0.028 (4)	0.037 (5)	0.003 (3)	0.006 (3)	−0.010 (4)
C11B	0.035 (5)	0.025 (7)	0.068 (10)	−0.010 (5)	0.020 (6)	−0.022 (6)
C8	0.0293 (14)	0.0243 (14)	0.0320 (15)	0.0063 (11)	0.0055 (12)	0.0006 (11)
C9	0.0258 (13)	0.0258 (14)	0.0213 (13)	0.0017 (11)	0.0022 (11)	−0.0025 (10)
C10	0.0251 (13)	0.0213 (13)	0.0187 (12)	−0.0004 (10)	0.0020 (10)	0.0010 (10)
N1	0.0279 (12)	0.0219 (12)	0.0223 (11)	0.0073 (9)	−0.0043 (9)	−0.0025 (9)
N2	0.0245 (11)	0.0215 (11)	0.0213 (11)	0.0035 (9)	0.0000 (9)	−0.0008 (9)
N3	0.0247 (11)	0.0251 (12)	0.0188 (11)	0.0003 (9)	−0.0008 (9)	−0.0020 (9)
S1	0.0239 (4)	0.0254 (4)	0.0245 (4)	0.0053 (3)	−0.0022 (3)	0.0000 (3)

### Geometric parameters (Å, °)

**Table:**

C1—C10	1.354 (4)	C7A—H7A	0.9800
C1—C8	1.493 (4)	C11A—H11A	0.9600
C1—S1	1.741 (3)	C11A—H11B	0.9600
C2—N3	1.358 (3)	C11A—H11C	0.9600
C2—C3	1.397 (4)	C6B—C7B	1.120 (9)
C2—S1	1.725 (3)	C6B—H6B1	0.9700
C3—C4	1.424 (4)	C6B—H6B2	0.9700
C3—C10	1.443 (4)	C7B—C11B	1.51 (2)
C4—N1	1.331 (3)	C7B—C8	1.616 (9)
C4—N2	1.351 (3)	C7B—H7B	0.9800
C5—C6A	1.489 (8)	C11B—H11D	0.9600
C5—C10	1.504 (4)	C11B—H11E	0.9600
C5—C6B	1.601 (7)	C11B—H11F	0.9600
C5—H5A	0.9700	C8—H8A	0.9700
C5—H5B	0.9700	C8—H8B	0.9700
C6A—C7A	1.424 (9)	C9—N3	1.323 (3)
C6A—H6A1	0.9700	C9—N2	1.335 (3)
C6A—H6A2	0.9700	C9—H9	0.9300
C7A—C8	1.503 (6)	N1—H1A	0.8600
C7A—C11A	1.556 (19)	N1—H1B	0.8600
			
C10—C1—C8	126.3 (2)	C7B—C6B—H6B2	105.5
C10—C1—S1	112.8 (2)	C5—C6B—H6B2	105.5
C8—C1—S1	120.9 (2)	H6B1—C6B—H6B2	106.1
N3—C2—C3	126.3 (2)	C6B—C7B—C11B	124.3 (10)
N3—C2—S1	122.3 (2)	C6B—C7B—C8	124.8 (6)
C3—C2—S1	111.4 (2)	C11B—C7B—C8	106.6 (11)
C2—C3—C4	114.6 (2)	C6B—C7B—H7B	96.9
C2—C3—C10	112.2 (2)	C11B—C7B—H7B	96.9
C4—C3—C10	133.2 (2)	C8—C7B—H7B	96.9
N1—C4—N2	116.6 (2)	C7B—C11B—H11D	109.5
N1—C4—C3	123.6 (2)	C7B—C11B—H11E	109.5
N2—C4—C3	119.7 (2)	H11D—C11B—H11E	109.5
C6A—C5—C10	112.9 (4)	C7B—C11B—H11F	109.5
C6A—C5—C6B	30.2 (4)	H11D—C11B—H11F	109.5
C10—C5—C6B	108.8 (3)	H11E—C11B—H11F	109.5
C6A—C5—H5A	109.0	C1—C8—C7A	112.0 (3)
C10—C5—H5A	109.0	C1—C8—C7B	107.3 (3)
C6B—C5—H5A	134.1	C7A—C8—C7B	31.6 (3)
C6A—C5—H5B	109.0	C1—C8—H8A	109.2
C10—C5—H5B	109.0	C7A—C8—H8A	109.2
C6B—C5—H5B	83.3	C7B—C8—H8A	135.6
H5A—C5—H5B	107.8	C1—C8—H8B	109.2
C7A—C6A—C5	124.2 (6)	C7A—C8—H8B	109.2
C7A—C6A—H6A1	106.3	C7B—C8—H8B	82.5
C5—C6A—H6A1	106.3	H8A—C8—H8B	107.9
C7A—C6A—H6A2	106.3	N3—C9—N2	128.1 (3)
C5—C6A—H6A2	106.3	N3—C9—H9	115.9
H6A1—C6A—H6A2	106.4	N2—C9—H9	115.9
C6A—C7A—C8	119.7 (5)	C1—C10—C3	112.1 (2)
C6A—C7A—C11A	122.4 (7)	C1—C10—C5	120.7 (2)
C8—C7A—C11A	111.5 (7)	C3—C10—C5	127.1 (2)
C6A—C7A—H7A	98.4	C4—N1—H1A	120.0
C8—C7A—H7A	98.4	C4—N1—H1B	120.0
C11A—C7A—H7A	98.4	H1A—N1—H1B	120.0
C7B—C6B—C5	127.1 (6)	C9—N2—C4	118.6 (2)
C7B—C6B—H6B1	105.5	C9—N3—C2	112.6 (2)
C5—C6B—H6B1	105.5	C2—S1—C1	91.45 (13)
			
N3—C2—C3—C4	1.6 (4)	C11B—C7B—C8—C1	−173.8 (11)
S1—C2—C3—C4	−179.64 (19)	C6B—C7B—C8—C7A	87.6 (14)
N3—C2—C3—C10	−179.1 (3)	C11B—C7B—C8—C7A	−69.8 (12)
S1—C2—C3—C10	−0.3 (3)	C8—C1—C10—C3	−179.9 (3)
C2—C3—C4—N1	177.7 (3)	S1—C1—C10—C3	0.1 (3)
C10—C3—C4—N1	−1.5 (5)	C8—C1—C10—C5	1.8 (5)
C2—C3—C4—N2	−3.1 (4)	S1—C1—C10—C5	−178.3 (2)
C10—C3—C4—N2	177.8 (3)	C2—C3—C10—C1	0.1 (3)
C10—C5—C6A—C7A	−13.7 (15)	C4—C3—C10—C1	179.3 (3)
C6B—C5—C6A—C7A	74.5 (12)	C2—C3—C10—C5	178.4 (3)
C5—C6A—C7A—C8	−2.4 (17)	C4—C3—C10—C5	−2.5 (5)
C5—C6A—C7A—C11A	−152.0 (12)	C6A—C5—C10—C1	14.0 (8)
C6A—C5—C6B—C7B	−85.2 (13)	C6B—C5—C10—C1	−18.1 (5)
C10—C5—C6B—C7B	18.3 (13)	C6A—C5—C10—C3	−164.1 (7)
C5—C6B—C7B—C11B	153.1 (15)	C6B—C5—C10—C3	163.8 (4)
C5—C6B—C7B—C8	−0.4 (19)	N3—C9—N2—C4	−0.7 (4)
C10—C1—C8—C7A	−17.7 (5)	N1—C4—N2—C9	−178.0 (3)
S1—C1—C8—C7A	162.3 (4)	C3—C4—N2—C9	2.7 (4)
C10—C1—C8—C7B	15.5 (6)	N2—C9—N3—C2	−0.8 (4)
S1—C1—C8—C7B	−164.5 (5)	C3—C2—N3—C9	0.3 (4)
C6A—C7A—C8—C1	17.3 (10)	S1—C2—N3—C9	−178.4 (2)
C11A—C7A—C8—C1	169.9 (8)	N3—C2—S1—C1	179.2 (2)
C6A—C7A—C8—C7B	−70.1 (10)	C3—C2—S1—C1	0.3 (2)
C11A—C7A—C8—C7B	82.5 (10)	C10—C1—S1—C2	−0.2 (2)
C6B—C7B—C8—C1	−16.5 (14)	C8—C1—S1—C2	179.7 (2)

### Hydrogen-bond geometry (Å, °)

**Table:**

*D*—H···*A*	*D*—H	H···*A*	*D*···*A*	*D*—H···*A*
N1—H1A···N2^i^	0.86	2.13	2.992 (3)	175
C7A—H7A···N2^ii^	0.98 (1)	2.47 (1)	3.400 (7)	158
C7B—H7B···S1^iii^	0.98 (1)	2.69 (1)	3.649 (1)	165

Symmetry codes: (i) −*x*, −*y*+2, −*z*; (ii) *x*, *y*−1, *z*; (iii) −*x*+1, −*y*+1, −*z*.
